# The Effect of the Use of Hearing Aids in Elders: Perspectives

**DOI:** 10.3390/audiolres12020017

**Published:** 2022-03-05

**Authors:** Daniele Monzani, Riccardo Nocini, Maria Teresa Presutti, Chiara Gherpelli, Federica Di Berardino, Silvia Ferrari, Gian Maria Galeazzi, Gaia Federici, Elisabetta Genovese, Silvia Palma

**Affiliations:** 1 Section of Otolaryngology and Audiology, Department of Diagnostics, Clinical and Public Health Medicine, University of Modena and Reggio Emilia, 41100 Modena, Italy; daniele.monzani@unimore.it (D.M.); 297209@studenti.unimore.it (M.T.P.); chiara.gherpelli@unimore.it (C.G.); gaia.federici@unimore.it (G.F.); elisabetta.genovese@unimore.it (E.G.); 2 Division of Otorhinolaryngology, Department of Surgery, Dentistry, Gynecology, and Pediatrics, University Hospital of Verona, Borgo Trento, Piazzale Aristide Stefani 1, 37126 Verona, Italy; riccardo.nocini@univr.it; 3 Audiology Unit, Department of Clinical Sciences and Community, Fondazione IRCCS Ca’ Granda Ospedale Maggiore Policlinico, Università degli Studi di Milano, 20021 Milan, Italy; federica.diberadino@policlinico.mi.it; 4 Section of Psychiatry, Department of Diagnostics, Clinical and Public Health Medicine, University of Modena and Reggio Emilia, 41100 Modena, Italy; silvia.ferrari@unimore.it (S.F.); gianmaria.galeazzi@unimore.it (G.M.G.); 5 Audiology, Primary Care Department, AUSL Modena, 41100 Modena, Italy

**Keywords:** presbycusis, fatigue, hearing aid, cognitive failures

## Abstract

Older adults with hearing loss have difficulties during conversation with others because an elevated auditory threshold reduces speech intelligibility, especially in noisy environments. Listening and comprehension often become exhausting tasks for hearing-impaired elders, resulting in social isolation and depression. The aim of the present study was to investigate the advantages of hearing aid use in relation to relief from listening-related fatigue, which is still controversial. Participants included a sample of 49 hearing-impaired elders affected by presbycusis for whom hearing aids were prescribed. The Modified Fatigue Impact Scale was used to assess cognitive, physical and psychosocial fatigue. The vitality subscale of the Short Form Health Survey 36 and a single item of the multi-dimensional Speech, Spatial and Quality Hearing Scale (“Do you have to put a lot of effort to hear what is being said in conversation with others?”) were also used. The Cognitive Failures Questionnaire was used to investigate daily errors related to lack of memory and reduced mindedness. Hearing aids rehabilitation resulted in improved speech intelligibility in competing noise, and a significant reduction in cognitive and psychosocial fatigue and listening effort in conversation. Vitality was also improved and a significant reduction in the Cognitive Failures Questionnaire scores was observed. Findings from the study indicate that the use of hearing aids in older impaired-listeners provide them not only with an increased auditory function but also with a reduction in listening-related fatigue and mindedness.

## 1. Introduction

Fatigue is very common in older people and interferes with daily activities, worsening their quality of life. Its prevalence increases with age [1] and also exists as a specific geriatric entity, not necessarily associated with physical or psychiatric conditions [2,3]. Despite its high incidence [4,5], it is a symptom that often remains an underestimated complaint.

For subjects with hearing impairment, the sensory loss results in a degradation of the quality of peripheral signals, which results in the requirement of increased cognitive resources to recognize relevant information from the speech stream, analyze context and recall prior phonological knowledge [6,7,8,9]. This shift from mostly bottom-up (signal-based) to mostly top-down (knowledge-based) auditory processing may increase the exertion of mental energy and is considered a result of both ageing and hearing loss [10,11].

Mental fatigue, in turn, is proved to be more detrimental to goal-directed (top-down) rather than stimulus-driven (bottom-up) attentive efforts, which potentially creates a self-reinforcing loop in hearing-impaired subjects [12,13]. Unfortunately, most studies evaluated listening effort and mental fatigue after a single session of speech perception under different laboratory conditions [14,15,16]. Similarly, results of experiments on the effectiveness of hearing aids in reducing listening effort and mental fatigue were limited to the duration of the tests [17,18].

On the contrary, mental fatigue as a chronic condition in hearing-impaired people should be more appropriately considered as a result of increased listening effort and cognitive load over a long period of time [19]. As a matter of fact, clinical evidence supporting this observation is accumulating in the medical literature [7,20,21], but is mainly limited to a working age population whereas investigations in the elderly and retired individuals are not well documented.

Besides this, mental fatigue in hearing-impaired older adults is also due to both depression and cognitive decline, which are frequently associated [22,23] and could per se account for increased exhaustion [24]. Anxiety is one of the most common late-life psychological symptoms [25] and anxious older people experience more fatigue than those without affective disturbance [26]. Consequently, the assessment of cognitive fatigue in elderly hearing-impaired listeners is potentially confounded by psychological symptoms. The purpose of this study was to assess fatigue in a cohort of hearing-impaired older people before and after the fitting of hearing aids, by attenuating the effect of these possible confounders.

## 2. Materials and Methods

The study cohort was composed of 46 older hearing-impaired subjects, receiving a first prescription for hearing aids at the Tertiary Audiological Centre of the University Hospital of Modena (Italy). Epidemiological data are presented in Table 1.

The study was approved by the regional (AVEN) Ethical Committee (n° 260/2009). Informed consent was obtained from all participants.

### 2.1. First Assessment

All subjects were aged ≥65 years and presented bilateral, symmetrical sensorineural hearing loss (i.e., no more than a 15 dB difference between ears in pure tone threshold at any octave frequency from 500 through 4000 Hz) and a downward sloping configuration of the audiogram for which no cause could be found other than presbycusis [27]. The use of ototoxic drugs, noise-exposure, smoking and alcohol abuse were also investigated and considered exclusion criteria. The study was conducted between 1 January 2012 and 31 December 2014.

Subjects underwent a first evaluation session including a collection of sociodemographic data and medical history. The otologic examination included otoscopy, pure tone audiometry, tympanometry and an acoustic reflex threshold test. Sensorineural hearing loss was confirmed by a normal tympanogram and by a speech-frequency pure tone average (PTA) of thresholds at 0.5, 1, 2, and 4 kHz in the better ear of above 15 dB [28].

Subjects selected to be included in the baseline assessment of pre-fitting functioning were then examined by two senior psychiatrists: the presence of a diagnosable mood, anxiety or psychotic disorder, which were ruled out by the administration of the Structural Clinical Interview (SCID-I) screening tool [29], and presence of any major medical and neurological disorders determined exclusion from the study.

Subjects admitted to the study after psychiatric evaluation were asked to fill in the following questionnaires:

- The single item of the multi-dimensional Speech, Spatial and Quality Hearing Scale (SSQ) by Gatehouse and Noble [30]: “Do you have to put a lot of effort to hear what is being said in conversation with others?”. Patients were invited to rate their answer on a numeric scale from 0 to 10, with lower values indicating greater effort.
- Modified Fatigue Impact Scale (MFIS) [31], Italian version. This 21-item multidimensional instrument includes physical, cognitive, and psychosocial subscales and assesses the impact of fatigue on a variety of daily activities. It was originally developed to assess fatigue in multiple sclerosis and later applied to other geriatric clinical populations [32]. Scoring is calculated by summing answers on a 4-point Likert-scale, and ranges between 0 and 84, with higher scores indicating a greater impact of fatigue on quality of life. Subscale scores for physical (9 items), cognitive (10 items), and psychosocial functioning (2 items) can also be generated by calculating the sum of the specific sets of items.
- The vitality subscale of the 36-item Medical Outcomes Study Short Form Health Survey (SF-36), Italian version [33]. It consists of four items and was designed to assess fatigue in healthy respondents from the general population and across chronic disease groups. Answers are rated on a 6-point Likert scale and the score is transformed onto a scale ranging from 0, indicating worst perception, to 100 that indicates best vitality.
- The Cognitive Failure Questionnaire (CFQ) [34], Italian version [35]. The CFQ is a 25-item self-report focusing on real-life cognitive performances and investigating daily errors (or lapses) related to deficit in memory, absent-mindedness and slips in action. Each item is a negatively phrased question rated on a 5-point scale ranging from 0 (never) to 4 (very often), giving a maximum score of 100 that indicates the worst result. Due to its high test–retest reliability, the CFQ is reliable in detecting variations in behavioral problems related to attentiveness and memory in everyday life before and after treatment [36].

Binaural, behind-the-ear (BTE) hearing aids were prescribed. All hearing aids were at the minimum feature settings with digital feedback reduction, digital noise reduction and automatically activated fixed directional systems. These systems automatically engage the directional microphone instead of the omnidirectional one when background noise is present, via signal processing decisions made for a given listening environment, and they employ a stationary polar pattern.

### 2.2. Final Assessment

About six months later, after optimization of amplification and acclimatization to hearing aids, the same subjects were asked to fill in the aforementioned questionnaires again and submitted to a simplified procedure of speech audiometry in aided and unaided conditions.

**Figure 1:**
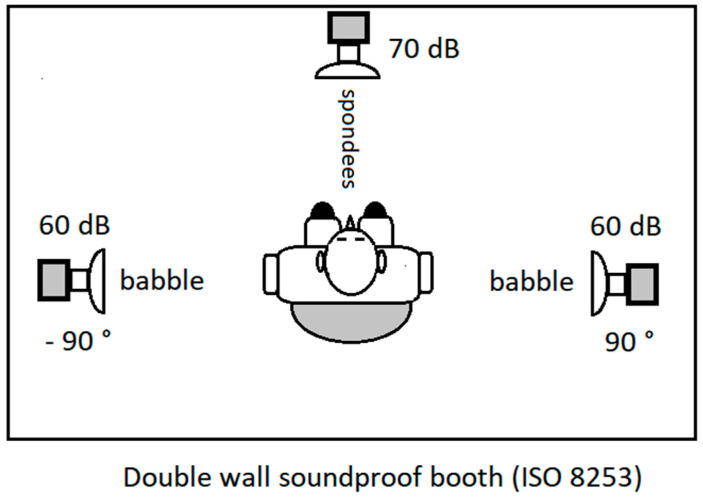
The figure shows the experimental setup in the double-wall soundproof booth.

Speech audiometry material contained 5 lists of 20 common two-syllable recorded words, the so-called spondees [37]. The patients were seated in a double wall booth and lists of words were delivered by a loudspeaker directly in front of the subject, one meter from their head at a fixed level of 70 dB (A). A ten-speaker multi-talker speech babble was used as the competing noise and delivered by two loudspeakers positioned at 90° degrees on each side of the patients (Figure 1). The spectrum of multi-talker babbles was speech shaped [38]. A signal-to-babble ratio of +10 dB [39,40] was adopted.

The number of words correctly repeated was computed as a percentage. An average use per day (expressed in hours) was obtained by data-logging. A single patient’s amplification and other technical parameters of the hearing aids were not assessed as variables in this study.

### 2.3. Statistical Analysis

Statistical analysis was performed using SPSS 20. Descriptive statistics are reported as mean, standard deviation of the means and minimum/maximum values. The Wilcoxon signed-rank test was the non-parametric statistical tool used to compare the repeated measurements on a single sample and a 0.05 level of statistical significance was adopted. Data are reported separately for patients with mild and moderate hearing loss. Since only three patients with severe hearing loss were recruited, they were excluded from the statistical analyses. Effect sizes for non-parametric variables were computed by dividing the test statistic z by the square root of the number of observations (n° of cases × 2) over the two time points according to the formula: r = Z/√n [41]. According to Cohen’s criteria r = 0.1 was considered a small effect size; = 0.3, a medium effect size; and = 0.5, a large effect size [42]. Data are reported separately for patients with mild and moderate hearing loss.

## 3. Results

The degree of hearing loss expressed in dB HL was mild (26 to 40) in 10 (21.7%) cases and moderate (41 to 55) in 36 (78.3%). The mean duration of daily use of hearing devices was 10.1 h (range 6–14, sd = 1.8), slightly less in comparison with previous reports on retired, over 65-year-old hearing-impaired individuals [43].

### 3.1. Patients with Mild Hearing Loss

All variables are presented in Table 2. The mean values, standard deviations, *Z*-value and asymptotic, two-tailed significance are reported. The PTA in the aided condition was significantly improved compared to the unaided one and had a large effect size (r = 0.65).

The improvement in the intelligibility of words was also significant (*p* < 0.005) and had a large effect size (r = 0.65). The CFQ score showed a significant improvement (*p* < 0.05) and the effect size was medium (r = 0.49). The single item of the SSQ “Do you have to make a lot of effort to hear what is being said in conversation with others?” showed a significant reduction in perceived listening effort after acclimatization to hearing aids (*p* < 0.05) and the effect size was large (r = 0.62)

The MFIS cognitive subscale scores indicated a remarkable improvement in perceived mental fatigue in hearing-impaired elders after acclimatization and use of hearing aids (*p* < 0.05) and the effect size was large (r = 0.54). A significant improvement was also observed in the psychosocial fatigue subscale (*p* < 0.05) with large effect size (r = 0.59). The physical subscale scores did not show any significant change at the final assessment (*p* > 0.05). Finally, the score of the vitality subscale (SF-36) significantly increased in patients with hearing aids (*p* < 0.05) and the effect size was medium (r = 0.49).

### 3.2. Patients with Moderate Hearing Loss

All variables are presented in Table 3. Additionally, in these subjects, the PTA in the aided condition and the intelligibility of words were significantly improved in comparison with the unaided one and the effect size was large (r = 0.65). The CFQ score exhibited a significant improvement and the effect size was medium (r = 0.36); similarly, the single item of the SSQ showed a significant decrease in perceived listening effort at final assessment (*p* < 0.005) and the effect size was medium (r = 0.38). The MFIS cognitive subscale scores also suggested significant improvement of perceived cognitive fatigue and the effect size was medium (r = 0.32). However, a remarkable improvement was observed in the psychosocial fatigue subscale (*p* < 0.001), but the effect size was medium (r = 0.45). The score of the vitality subscale (SF-36) significantly improved at final assessment and the effect size was medium (r = 0.39).

## 4. Discussion

Hearing loss is a chronic and often worsening type of sensory deprivation, with related neuropsychological consequences when left untreated [22]. It has been established that hearing aid use improves adults’ hearing-related quality of life by reducing the psychological, social, and emotional effects of hearing impairment [22] and, in addition, a large epidemiological, cross-sectional survey showed that older subjects with hearing aids reported much less fatigue in communication with others than those who did not wear them [21].

This study confirmed that the use of auditory devices allows for better word comprehension in competing noise in elders affected by both mild and moderate hearing loss. It should be noted that signal and noise in the experimental setting were spatially separated so as to involve a lower cognitive load on perceptual and cognitive resources for speech understanding [44].

Moreover, the signal-to-babble ratio of 10 dB is probably more favorable for aided elders than those encountered in realistic sound scenarios [17].

Listening-related fatigue is a common complaint among elders with presbycusis and is also thought to depend on cognitive overload, which is required to compensate for hearing impairment. It is known that cognitive load and listening effort are reduced by use of hearing aids in elders, even when they do not significantly improve speech recognition tests in laboratories [45]. The perception of listening effort of all hearing impaired elderly patients was alleviated by hearing aids, as demonstrated by the change in score of the single item of the SSQ, which showed a significant increase.

In this sample, no amelioration of physical fatigue was reported. A possible explanation is that auditory deprivation generates a high demand on cognitive resources that could be alleviated by hearing aids but is not related to functional limitation, pain, or muscular weakness, causing physical fatigue such as in patients with cancer [46]. Physical and mental fatigue, therefore, seem to be independent variables in hearing-impaired older listeners, outlining the necessity to assess fatigue and its changes after aural rehabilitation using a multidimensional approach. At the same time, hearing-impaired elders probably perceive themselves as fatigued by social activities, maybe depending on poor individual coping strategies and problem-solving abilities [47] or, more simply, because it is too exhausting for them to hold a conversation with others requiring a lot of effort. This fatigue is also relieved by the use of hearing aids, regardless of the degree of hearing loss, as our study shows.

Additionally, patients’ perception of vitality was significantly increased after hearing aids use; the magnitude of the effect was considerable and the minimally important difference (5 points) for the scale was reached [48] in subjects with both mild and moderate hearing loss. This result seems in apparent contrast with a recent experience, which found no relief from a low level of vitality by the use of hearing aids in older hearing-impaired people [49]. This discrepancy may be due to the fact that, in the aforementioned study, vitality was identified as an emotional construct strictly depending on depression and anxiety, conditions which were screened out in our study.

The fact that the CFQ score was significantly improved by the use of hearing aids can be interpreted by the finding that a higher score is generally associated with a better selective attention, maybe due to a more efficient active inhibition of distractors [50]. Since selective attention is necessary to focus on the desired auditory object and filter out competing sound sources [51], a partial recovery of degraded peripheral auditory processing could facilitate this process, redirect focused attention to other daily demanding tasks [52] and, consequently, alleviate cognitive failures. Moreover, Boksen [12] showed that fatigued individuals have defective sustained attention, and difficulties in ignoring irrelevant information and correcting their mistakes. If this is the case, common sense seems to indicate that hearing-impaired elders could benefit from hearing aid use because of a sort of re-allocation of cognitive resources and/or a cognition-sparing mechanism. Criticism may arise from the study design that did not include objective cognitive tests to assess selective attention before and after aural rehabilitation, but it should be considered that those tests are usually carried out in comfortable laboratories and limited to only one or two points in time.

On the other hand, the strength of individuals’ reports of attention failures over a relatively long time interval (i.e., six months before CFQ administration) provides the examiners with a better understanding of how cognitive resources support hearing-impaired elders in everyday life and improve appreciation of underestimated cognitive issues related to sensory deprivation.

Research into the benefits of hearing aid signal processing has focused its effects on laboratory speech perception tests, evaluating changes in intelligibility and listening effort. However, laboratory speech perception tests have generally failed to capture the complexity of the impairment in understanding everyday life conversation, overlooking cognitive and listening-related fatigue. Further analysis and better understanding of the dimension of fatigue seems particularly important in view of recent findings on fatigue and vigor in patients with hearing impairment, which indicates that fatigue does not directly correlate with the degree of hearing loss [53], but with patients’ perception of hearing disability, thus highlighting the importance of non-auditory and individual factors in this area [19].

## 5. Conclusions

This study shows that susceptibility to both cognitive fatigue and cognitive failures in elders affected by both mild and moderate hearing loss are alleviated by the use of hearing aids, in the absence of major psychiatric disorders. These results also suggest that it would be useful to include a multidimensional fatigue scale and a questionnaire to assess everyday life cognitive lapses in the standard assessment of elder subjects. This could be helpful in providing hearing-impaired older subjects with feedback on their sensory frailty and to improve it through hearing rehabilitation.

## Figures and Tables

**Table 1:** Demographic data of the entire sample.

Overall Hearing-Impaired Patients (*n* = 46)				
Males (n = 25) Females (*n* = 21)				
	Mean	Standard Deviation	Minimum	Maximum
Age (years)	73.0	5.8	64	78

**Table 2:** Means and standard deviations (SD) of the hearing threshold (PTA), word recognition score (WRS), the Cognitive Failure Questionnaire (CFQ), the single item of the SSQ, and the Modified Fatigue Impact Scale (MFIS) subscales scores at the first and final assessment after patients’ acclimatization to hearing aids use are presented. The Wilcoxon signed rank test is expressed by *Z*-value and Asymptotic Significance (two tails). (**) = *p* < 0.005, (*) = *p* < 0.005.

	First Examination		Final Examination			
	(Unaided)		(Aided)		Wilcoxon’s Signed-Rank Test	
	Mean	SD	Mean	SD	*Z*-Value	Asymp. Sig. (Two Tails)
PTA	37.1	1.6	24.3	2.7	−2.94	0.003 **
WRS	64.4	7.8	75.5	7.6	−2.93	0.004 **
CFQ.	44.2	4.9	39.5	6.6	2.2	0.028 *
SSQ (single item)	3.8	1.1	5.4	1.9	−2.8	0.005 *
MFIS						
Cognitive subscale	7.3	1.2	6.1	1.0	2.43	0.015 *
Psychosocial subscale	5.4	1.3	3.1	1.3	2.66	0.008 *
Physical subscale	5.5	1.7	4.5	1.1	1.49	0.135
Vitality scale (SF-36)	51.4	5.3	58.3	8.9	−2.22	0.026 *

**Table 3:** Means and standard deviations (SD) of the hearing threshold (PTA), word recognition score (WRS), the Cognitive Failure Questionnaire (CFQ), the single item of the SSQ, and the Modified Fatigue Impact Scale (MFIS) subscales scores at the first and final assessment after patients’ acclimatization to hearing aids use are presented. The Wilcoxon signed rank test is expressed by *Z*-value and Asymptotic Significance (two tails). (**) = *p* < 0.005, (*) = *p* < 0.005.

	First Examination		Final Examination			
	(Unaided)		(Aided)		Wilcoxon’s Signed-Rank Test	
	Mean	SD	Mean	SD	*Z*-Value	Asymp. Sig. (Two Tails)
PTA	45.4	3.9	30.7	3.4	−5.16	<0.001 (**)
WRS	50.8	3.4	68.1	8.2	−5.89	<0.001 (**)
CFQ.	61.2	7.4	41.7	9.0	−3.1	0.002 (**)
SSQ (single item)	4.4	1.4	5.7	1.5	−3.22	0.001 (**)
MFIS						
Cognitive subscale	8.6	2.1	7.1	2.5	−2.75	0.006
Psychosocial subscale	5.4	1.8	3.5	1.4	−3.8	<0.001 (**)
Physical subscale	4.8	0.9	4.8	0.9	−0.227	0.820
Vitality scale (SF-36)	51.6	5.6	56.6	7.5	−3.32	0.001 (**)

## Data Availability

The data presented in this study are available on request from the corresponding author, due to privacy restrictions.
